# Fractures of the Ribs, and of Their Cartilages

**Published:** 1858-03

**Authors:** Frank H. Hamilton


### BUFFALO MEDICAL JOURNAL

AND

### MONTHLY REVIEW.

VOL. 13. MARCH, 1858. NO. 10.

### ORIGINAL COMMUNICATIONS.

ART. I.—*Fractures of the Ribs, and of their Cartilages.* By Frank H. Hamilton, M. D.

### § I. *Fractures of the Ribs.*

Fractures of the ribs, observed more often than fractures of the sternum, are rare as compared with fractures of other long bones.

In my records only twelve patients are reported as having had broken ribs; but as in several of the cases two or more ribs were broken at the same time, the total number of fractures is about thirty. If, however, I had always accepted of the diagnosis made by other surgeons the number would have been much greater, since I have been repeatedly assured that the ribs were broken where, upon the most rigid examination, no evidence, beyond the existence of a severe pain and of difficult respiration, has been presented to me.

*Etiology.* The force requisite to break the ribs is scarcely less than what is requisite to break the sternum; and in childhood and infancy it is sometimes almost impossible to break them, so that children and even adults are often crushed and killed outright, where, although the pressure has been directly upon the thorax, the ribs have resumed their positions, and have been found not to be broken. I have met with several examples of this kind.

In old age, the cartilages ossify and the ribs themselves suffer a gradual atrophy, which renders them much more liable to break.

The most common causes are direct blows, of very great force, in consequence of which sometimes the fragments are not only broken but more or less forced inwards; occasionally they are the result of counter-strokes, and then the fragments, if they deviate at all from their natural position, are salient outwards: a species of fracture which I have not met with so often.

Malgaigne has collected eight examples of fractures of the ribs produced by muscular action, by the beating of the heart, &c., all of which occurred upon the left side. It is believed, however, that in all of these cases the ribs had previously become atrophied, and perhaps undergone other changes in their structure, rendering them liable to fracture from the action of trivial causes.

*Pathology, Seat, &c.* The fourth, fifth, sixth and seventh ribs are most liable to be broken; the upper ribs, and especially the first rib, being so well protected in various ways as to greatly diminish their liability, while the loose and floating condition of the last two ribs gives them an almost complete exemption.

In my own cases I have found the first, second, third and fourth ribs, each broken once; the fifth, six times; the sixth, seven times; the seventh, six times; the eighth and ninth, once; and the tenth, twice.

Fourteen were broken through their anterior thirds, generally at or near the junction of the cartilages with the ribs; five through their middle thirds; and nine through their posterior thirds. Malgaigne has noticed, also, contrary to the general opinion of surgeons, that the ribs are most often broken in their anterior thirds, whether the cause has been a direct or a counter blow.

The direction of the fracture is generally transverse or slightly oblique: sometimes it is quite oblique. It is often compound; and in a few instances I have found it comminuted or multiple. Where the fracture is compound it is rendered so generally by the fragments having penetrated the lungs and not by a tegumentary wound. In only six of the twelve cases seen by me, has the fracture been uncomplicated with fractures or dislocations of other bones.

Displacement cannot occur in the direction of the axis of the bone unless several ribs be broken at the same time. The fragments are, therefore, either not at all displaced, or they fall inwards towards the cavity of the chest, or outwards, or very slightly downwards, in the direction of the intercostal spaces. Sometimes the rib moves a little upon its own axis.

*Prognosis.* Death occurs sooner or later in a pretty large proportion of the cases in which the ribs have been broken; yet not often as a direct consequence of the fracture, but only as a result of the injury inflicted upon the viscera of the chest, or of other injuries received at the same moment. The violent compression of the heart and lungs have frequently produced death, and sometimes, as I have more than once seen, almost immediately; or the patients have succumbed at a later period to acute pneumonitis.

Lonsdale saw a case in which the body of a man having been traversed by the wheel of a wagon, eight ribs were broken, and death having followed most immediately, the autopsy disclosed a rent in the left auricle of the heart, produced by one of the broken ribs. *Lonsdale*, p. 258.

South says there is such a specimen in St. Thomas Hospital. *Chelius*, v. i. p. 599.

Dupuytren reports a similar case. The same surgeon has also seen several deaths produced by the emphysema, independent of the fracture, two of which are particularly described in his Clinical Lectures, (pp. 79-80.)

Amesbury has seen a case of death from rupture of the intercostal artery, where there was no injury of the lungs. *Amesbury on Frac.*, v. ii, p. 612.

In several instances observed by me, patients have suffered from pains in the side, occasionally from cough, &c., after the lapse of two or more years, and I suspect it is no uncommon thing for these injuries to entail some such permanent disability, but which is a consequence rather of the injury to the viscera of the chest than of any condition of the broken ribs themselves.

In general, simple fractures of the ribs unite in from twenty-five to thirty days. Malgaigne has seen one case of non-union; Huquier met with another upon the cadaver, in which a complete false joint existed, furnished with a capsule and lined with synovial membrane, (*Malgaigne*, p. 435.) Eve, of Nashville, Tenn., saw a case of non-union occasioned probably by a caries or necrosis of the bone, since it was accompanied with a discharge of matter, and in which a removal of the ends of the fragments resulted promptly in a cure of the sinus, (*New York Jour. of Med.*, v. xvi, p. 136,) and Samuel Cooper says there is a specimen in the museum of the University College, of a fracture of six ribs, where the fragments are only connected by a fibrous or ligamentous tissue. (*S. Coop. Surgery*, v. ii, p. 321.)

The union generally occurs with only a slight degree of displacement.

After the union is completed, even where there is no displacement, a certain amount of ensheathing callus may generally be felt at the point of fracture. Of five cases which I have carefully examined after recovery, in only one instance was I unable to detect any irregularity at this point. I have in my cabinet nine specimens of fractured ribs, in four of which the ensheathing callus is completely formed, but the fragments are in perfect apposition: in one apposition is preserved, but there is no ensheathing callus; and the remaining four, all occurring in the same person, are united with displacement, but without a proper ensheathing callus.

In some specimens I have observed sharp spiculæ of bone or osteophytes extending along the course of the intercostal muscles from one rib to the other, forming a species of anchylosis between their adjacent margins.

*Symptomatology.* Acute pain, referred especially to the point of fracture, and producing great embarrassment in the respiration, with crepitus, are the most common indications of a fracture. The pain and embarrassed respiration are, however, far from being diagnostic, since they are often present in an equal degree when the walls of the chest have only been severely contused.

The crepitus, also, is often difficult to detect, owing to the thickness of the muscular coverings, or to the amount of fat upon the body, or to the fracture having occurred perhaps directly underneath the mammæ in the female. In three instances, where the presence of emphysema rendered the existence of a fracture quite certain, I have been unable immediately after the accident to discover crepitus.

The crepitus may be discovered sometimes by pressing gently upon the seat of fracture, or by applying the ear or the stethescope over this point while the patient attempts a full inspiration, or coughs; or we may press upon the front of the chest with one hand, while the fingers of the other hand rest upon the fracture.

Occasionally the patient has felt the bone break, and very often he feels or hears the crepitus after it is broken and will himself indicate very clearly the point of fracture.

At the same time that we detect crepitus we are able also to discover motion in the fragments, but I have once or twice discovered preternatural mobility without crepitus.

Emphysema, which is almost certainly indicative of a fracture, is present in a pretty large proportion of cases. It has been observed by me in seven out of twelve cases: generally it did not extend over more than two or three square feet of surface; but in one instance it finally extended over nearly the whole body. It is remarkable, however, that in only two of these seven cases did the patients expectorate blood, and then in a very small quantity, and not until the second or third day.

Desault observes that emphysema rarely succeeds to fractures of the ribs; an observation which, as will be seen, my experience does not at all confirm.

*Treatment.* In simple fractures, where there is no displacement, or where the displacement is only moderate, the chest may be enclosed with a broad belt or band, as we have already directed in case of fracture of the sternum: provided always that it is not found to increase instead of diminishing the patient's sufferings. Some patients cannot tolerate this confinement at all, while with a majority, although it is at first uncomfortable and oppressive, after an hour or two it affords them great relief from the distressing pain, and they will not consent to have it removed even for a moment. In nearly all cases of comminuted, or multiple fracture, it is inadmissable, on account of its tendency to force the pieces inwards.

Hannay, of England, has suggested the use of adhesive straps as a substitute for the cotton or flannel band; the several successive pieces being imbricated upon each other until the whole chest is covered.* The same objection holds to this mode of dressing as to a similar mode of dressing a broken clavicle, which has been recently recommended. It will certainly become loosened after a few hours, by the slight but uninterrupted play of the ribs.

The forearm ought also to be brought across the chest at a right angle with the arm, and secured in this position with a moderately tight bandage or a sling, so as to prevent any motion in the pectoral muscles.

As to position, the patient generally prefers to sit up, or he chooses a position only partly reclining upon his back; but there is no positive rule to observed in this matter, except that such a position shall be chosen as shall prove most comfortable to the patient.

If the fragments are salient outwards, the fracture having been produced by a counter stroke, they may be reduced by pressing gently upon them from without. If, on the contrary, the fragments are salient inwards, they will be found, in a great majority of cases, to have resumed their positions

American Jour. Med. Sci', vol, xxxix, p. 198. From Lon. Med. Gaz., Nov., 1845.

spontaneously or through the natural actions of respiration; but if they have not, it will be exceedingly difficult to restore them. Possibly it may be accomplished by pressing forcibly upon the front of the chest, or upon the anterior extremity of the broken rib; yet if the fragments are comminuted, and the ends are much driven in, this method will avail little or nothing. In such cases several surgeons have recommended that we should cut down to the bone and elevate the fragments, but Rossi alone claims to have actually put the suggestion into practice.

No doubt, if the necessity was urgent, this method might be successfully adopted; or, instead of cutting down to the broken rib, we might even seize the fragment with a hook, as suggested by Malgaigne, or, what in some cases might be even more convenient, with a pair of forceps constructed with long teeth, obliquely set upon a firm shaft. Yet the exigency which will demand a resort to any of these measures will be exceedingly rare.

In no case do I attach any value or importance to the advice given by Petit, that we shall place a compress upon the front of the chest, underneath the bandage, in order to reduce the fragments, or to retain them in place after reduction. Lisfranc, who advocated this method, claimed that its advantage consisted in the increased length which was thus given to the antero-posterior diameter of the chest, and the consequent accumulation of pressure from the encircling band, in this direction.* The mechanical law is no doubt correctly stated, but its value in practice is two inconsiderable to deserve much consideration.

The emphysema generally demands no especial attention, since it is usually too limited to occasion inconvenience, and when more extensive it generally disappears spontaneously after a few days, or a few weeks at most. The advice given by some surgeons, that we ought in these cases to cut down to the pleural cavity so as to allow the air to escape freely through the incision, seems thus far to have resthed its reputation upon a more than doubtful theory rather than upon any testimony of experience. Abernethy alone, so far as I know, has actually made the experiment, and his patient died.

Dupuytren, in the two cases already alluded to, bled the patients and applied resolvent liquids, with rollers; he also made incisions with the lancet at various points of the body, more or less remote from the seat of fracture, a practice, however, in which he confesses he has no confidence whatever. These patients both died.

---

* Rankin's Abstract, vol. ii, p. 204, from Gaz. des Hôpitaux, July 8, 1845.

Dr. Stedman, of the Massachusetts General Hospital, has reported the case of a man aged 69, of intemperate habits, who, in addition to a fracture of one of his ribs, had also a dislocation of the outer end of the clavicle. The emphysema commenced immediately and reached its acme on the twenty-second day. At this time it had extended over his whole body; his eyes were closed and he breathed with great difficulty: but on the forty-fifth day, the emphysema had entirely disappeared, and he was dismissed cured. The treatment consisted chiefly in the free internal use of stimulants and in the application of bandages; but the bandages soon became disarranged, and after a few days they were entirely laid aside.*

In the case of my own patient, where the emphysema was almost equally extensive, the patient recovered after a few weeks, under the use of a simple diet, and without any special medication whatever.

### § II. *Fractures of the Cartilages of the Ribs.*

Boyer was incorrect when he said that the cartilages of the ribs could not be broken until they were ossified. They are often broken when there is no ossification, at the same time that the ribs themselves are broken. Sometimes they are broken alone. Not unfrequently, also, the separation takes place at the precise point of junction between the two.

Pyper relates a case in which the sternum was broken in a man aged 25 years, and also the cartilages of the sixth, seventh and eighth ribs of the right side, as was proven by the autopsy, yet the ribs were not ossified. The vena cava ascendens was also ruptured by the force of the compression.†

*Etiology.* The causes are the same which produce fractures of the ribs, yet it is generally understood that it will require greater force, and that consequently the injury done to the viscera of the thorax will be more complicated and intense.

In the reports of the Massachusetts General Hospital, an account is given of the case of a man aged 30, who was crushed by the fall of a heavy weight upon his body, and who died after about sixty hours. An examination after death, revealed a fracture of the cartilages of the third and fourth ribs, with a laceration of the intercostal muscles to such an extent that a hernia of the lungs had occurred at this point. This hernia had been discovered and

---

* Boston Med. & Surg. Jour., vol. lii, p. 316.

† Rankin's Abstract, vol. i, p. 147, from The Lancet, Oct., 1844.

recognized, by Dr. Warren, soon after the accident occurred; the protrusion being at that time as large as the clenched fist and regularly rising and falling with each movement of respiration. It was accompanied, also, with a moderate emphysema.*

*Pathology.* The fracture is clean and vertical, or transverse; never irregular or oblique. The direction of the displacement varies as in fractures of the ribs, but the anterior or sternal fragment is generally found in front of the posterior or spinal.

Union takes place in these fractures, not through the medium of cartilage, but of bone. Sometimes the new bone being deposited only between the ends of the fragments, in the form of a thin plate, and at other times, it is formed around the fragments as well as between them. The latter of these two processes has been most frequently observed. The ensheathing callus appears to be supplied by the perichondrium, while the experiments of Dr. Redfern render it probable that the intermediate callus may result from a conversion, or transformation, of the adjacent cartilaginous surfaces. Paget remarks, also, that the ossification extends to the parts of the cartilage immediately adjacent to the fracture.

I do not know that any observations have been made upon the repair of these cartilages in very early life, and it is possible that the process may differ from this which has been described as it has been observed in the adult.

*Treatment.* The treatment need not differ from that already recommended for fractured ribs.

* Boston Med. and Surg. Jour., vol. xxviii, p. 158.

